# 2,4-Dinitro­benzaldehyde hydrazone

**DOI:** 10.1107/S1600536808007514

**Published:** 2008-03-29

**Authors:** Nan Zhang, Ruji Wang, Chunyan Tan, Yuyang Jiang, Yufen Zhao

**Affiliations:** a Key Laboratory of Bioorganic Phosphorus Chemistry & Chemical Biology, Ministry of Education, Department of Chemistry, Tsinghua University, Beijing 100084, People’s Republic of China; b Department of Chemistry, Tsinghua University, Beijing 100084, People’s Republic of China; c Key Laboratory of Chemical Biology, Guangdong Province, Graduate School at Shenzhen, Tsinghua University, Shenzhen 518055, People’s Republic of China; d School of Medicine, Tsinghua University, Beijing 100084, People’s Republic of China

## Abstract

The title compound, C_7_H_6_N_4_O_4_, plays an important role in the synthesis of biologically active compounds. The planar hydrazone group is oriented at a dihedral angle of 8.27 (3)° with respect to the benzene ring. In the crystal structure, inter­molecular N—H⋯O and N—H⋯N hydrogen bonds link the mol­ecules.

## Related literature

For related literature, see: Allen *et al.* (1987 ▶); Chaulk *et al.* (2007 ▶); Kawakami *et al.* (2000 ▶); Moreno-Mañas *et al.* (2001 ▶).
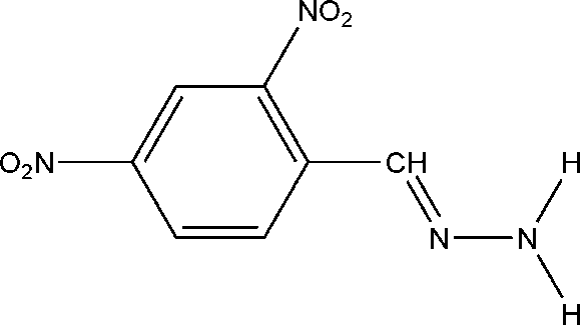


## Experimental

### 

#### Crystal data

C_7_H_6_N_4_O_4_
*M* *_r_* = 210.16
Triclinic, 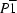

*a* = 4.5839 (7) Å
*b* = 9.6840 (16) Å
*c* = 9.9287 (15) Å
α = 90.785 (12)°
β = 96.149 (11)°
γ = 98.955 (13)°
*V* = 432.66 (12) Å^3^
*Z* = 2
Mo *K*α radiation
μ = 0.14 mm^−1^
*T* = 295 (2) K
0.4 × 0.3 × 0.2 mm

#### Data collection

Bruker P4 diffractometer
Absorption correction: none
2238 measured reflections
1616 independent reflections
1160 reflections with *I* > 2σ(*I*)
*R*_int_ = 0.026
3 standard reflections every 97 reflections intensity decay: none

#### Refinement

*R*[*F* ^2^ > 2σ(*F* ^2^)] = 0.048
*wR*(*F* ^2^) = 0.116
*S* = 1.07
1616 reflections
136 parameters
H-atom parameters constrained
Δρ_max_ = 0.16 e Å^−3^
Δρ_min_ = −0.18 e Å^−3^

### 

Data collection: *XSCANS* (Bruker, 1997 ▶); cell refinement: *XSCANS*; data reduction: *XSCANS*; program(s) used to solve structure: *SHELXTL* (Sheldrick, 2008 ▶); program(s) used to refine structure: *SHELXTL*; molecular graphics: *SHELXTL*; software used to prepare material for publication: *SHELXTL*.

## Supplementary Material

Crystal structure: contains datablocks I, global. DOI: 10.1107/S1600536808007514/fj2106sup1.cif

Structure factors: contains datablocks I. DOI: 10.1107/S1600536808007514/fj2106Isup2.hkl

Additional supplementary materials:  crystallographic information; 3D view; checkCIF report

## Figures and Tables

**Table 1:** Hydrogen-bond geometry (Å, °)

*D*—H⋯*A*	*D*—H	H⋯*A*	*D*⋯*A*	*D*—H⋯*A*
N1—H1*B*⋯O1^i^	0.90	2.52	3.305 (3)	146
N1—H1*C*⋯N2^ii^	0.90	2.34	3.123 (4)	146

Symmetry codes: (i) 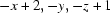; (ii) 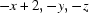.

**Fig. 1.**
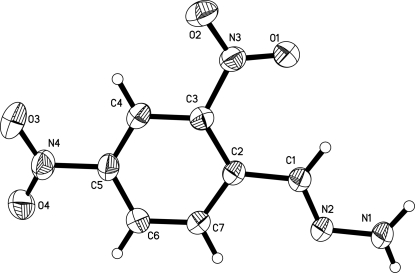
Perspective drawing of the title compound, with the atomic numbering scheme. Displacement ellipsoids are shown at the 35% probability level.

**Fig. 2.**
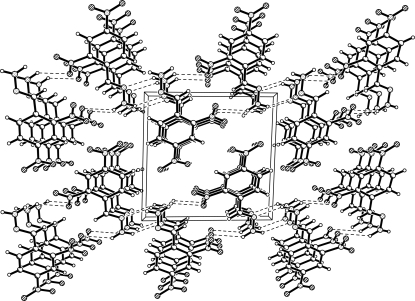
The unit cell packing of the title compound, viewed along the a direction.

## supplementary crystallographic information

### Comment

Benzaldehyde hydrazone and its analogues are important intermediates in heterocyclic chemistry, and they have been widely used for the synthesis of biologically active compounds such as [1,2,4]triazino[6,5-*f*]quinolines, pyrazolo[3,4-*f*]quinolines (Kawakami *et al.*, 2000), 1,3-dithiol-2-ylidene derivatives (Moreno-Mañas *et al.*, 2001), and oligo-RNAs with photocaged adenosine 2'-hydroxyls (Chaulk *et al.*, 2007). Here we report the synthesis and crystal structure of a nitro-analogue: 2,4-dinitrobenzaldehyde hydrazone. The molecule of the title compound (Fig. 1) contains a benzene ring, a hydrazone chain and two nitryl groups. Most of the bond lengths and angles are within normal ranges (Allen *et al.*, 1987). Because of the pi-pi conjugation and two nitryl groups electron withdrawing effect, the distance of C=N bond (1.282 (3) Å) is obviously shorter than that of the normal range (1.34–1.38 Å). The molecule is essentially planar, with a dihedral angle of 8.27° between the hydrazone group and the benzene ring. In the crystal structure, the molecules are linked by intermolecular N—H···O and N—H···N hydrogen bonds (Table 1, Fig. 2), which seem to be effective in the stabilization of the structure.

### Experimental

2,4-Dinitrobenzaldehyde (1.96 g, 10 mmol) was dissolved in 100 ml absolute ethanol, after which hydrazine hydrate (0.96 ml, 20 mmol) was added. The mixture was stirred at about 353 K for 5 h. The solution was cooled and kept at about 279 K overnight. Brown powder was collected by filtration (1.41 g, yield 67%) and then single crystals suitable for X-ray measurements were obtained by recrystallization from ethanol.

### Refinement

All non-H atoms were refined anisotropically. All H atoms were placed in calculated positions, with N–H = 0.9 Å and C–H = 0.93 Å. Final difference Fourier maps showed the highest and lowest electron densities of 0.160 and -0.177 e Å^-3^, respectively.

### Crystal data

**Table:**

C_7_H_6_N_4_O_4_	*Z* = 2
*M**_r_* = 210.16	*F*_000_ = 216
Triclinic, *P*1	*D*_x_ = 1.613 Mg m^−^^3^
Hall symbol: -P 1	Mo *K*α radiation λ = 0.71073 Å
*a* = 4.5839 (7) Å	Cell parameters from 39 reflections
*b* = 9.6840 (16) Å	θ = 5.9–12.5º
*c* = 9.9287 (15) Å	µ = 0.14 mm^−^^1^
α = 90.785 (12)º	*T* = 295 (2) K
β = 96.149 (11)º	Prism, yellow
γ = 98.955 (13)º	0.4 × 0.3 × 0.2 mm
*V* = 432.66 (12) Å^3^	

### Data collection

**Table:**

Bruker P4 diffractometer	*R*_int_ = 0.027
Radiation source: fine-focus sealed tube	θ_max_ = 25.5º
Monochromator: graphite	θ_min_ = 2.1º
*T* = 295(2) K	*h* = −5→1
ω scans	*k* = −11→11
Absorption correction: none	*l* = −11→11
2238 measured reflections	3 standard reflections
1616 independent reflections	every 97 reflections
1160 reflections with *I* > 2σ(*I*)	intensity decay: none

### Refinement

**Table:**

Refinement on *F*^2^	Secondary atom site location: difference Fourier map
Least-squares matrix: full	Hydrogen site location: inferred from neighbouring sites
*R*[*F*^2^ > 2σ(*F*^2^)] = 0.048	H-atom parameters constrained
*wR*(*F*^2^) = 0.116	*w* = 1/[σ^2^(*F*_o_^2^) + (0.001*P*)^2^ + 0.38*P*] where *P* = (*F*_o_^2^ + 2*F*_c_^2^)/3
*S* = 1.07	(Δ/σ)_max_ < 0.001
1616 reflections	Δρ_max_ = 0.16 e Å^−^^3^
136 parameters	Δρ_min_ = −0.18 e Å^−^^3^
Primary atom site location: structure-invariant direct methods	Extinction correction: none

### Special details

**Table:**

Geometry. All e.s.d.'s (except the e.s.d. in the dihedral angle between two l.s. planes) are estimated using the full covariance matrix. The cell e.s.d.'s are taken into account individually in the estimation of e.s.d.'s in distances, angles and torsion angles; correlations between e.s.d.'s in cell parameters are only used when they are defined by crystal symmetry. An approximate (isotropic) treatment of cell e.s.d.'s is used for estimating e.s.d.'s involving l.s. planes.
Refinement. Refinement of *F*^2^ against ALL reflections. The weighted *R*-factor *wR* and goodness of fit *S* are based on *F*^2^, conventional *R*-factors *R* are based on *F*, with *F* set to zero for negative *F*^2^. The threshold expression of *F*^2^ > σ(*F*^2^) is used only for calculating *R*-factors(gt) *etc*. and is not relevant to the choice of reflections for refinement. *R*-factors based on *F*^2^ are statistically about twice as large as those based on *F*, and *R*- factors based on ALL data will be even larger.

### Fractional atomic coordinates and isotropic or equivalent isotropic displacement parameters (Å^2^)

**Table:**

	*x*	*y*	*z*	*U*_iso_*/*U*_eq_	
O1	0.7840 (6)	0.1234 (3)	0.5319 (2)	0.0887 (8)	
O2	0.4576 (6)	0.2314 (2)	0.6030 (2)	0.0798 (7)	
O3	0.0003 (6)	0.5548 (2)	0.3362 (3)	0.0872 (8)	
O4	0.1241 (6)	0.5961 (3)	0.1351 (3)	0.0946 (9)	
N1	0.9783 (6)	−0.0870 (3)	0.1463 (3)	0.0758 (8)	
H1B	1.0167	−0.1364	0.2204	0.091*	
H1C	1.0581	−0.1043	0.0700	0.091*	
N2	0.8489 (5)	0.0275 (2)	0.1456 (2)	0.0578 (6)	
N3	0.5907 (6)	0.1955 (2)	0.5116 (2)	0.0569 (6)	
N4	0.1273 (6)	0.5291 (3)	0.2381 (3)	0.0675 (7)	
C1	0.7626 (6)	0.0654 (3)	0.2571 (3)	0.0520 (7)	
H1A	0.7984	0.0169	0.3358	0.062*	
C2	0.6075 (6)	0.1858 (3)	0.2585 (3)	0.0471 (6)	
C3	0.5165 (6)	0.2445 (3)	0.3752 (3)	0.0470 (6)	
C4	0.3552 (6)	0.3540 (3)	0.3687 (3)	0.0518 (7)	
H4A	0.2918	0.3885	0.4466	0.062*	
C5	0.2906 (6)	0.4105 (3)	0.2464 (3)	0.0534 (7)	
C6	0.3755 (6)	0.3582 (3)	0.1277 (3)	0.0580 (7)	
H6A	0.3302	0.3978	0.0448	0.070*	
C7	0.5269 (6)	0.2473 (3)	0.1360 (3)	0.0561 (7)	
H7A	0.5791	0.2108	0.0565	0.067*	

### Atomic displacement parameters (Å^2^)

**Table:**

	*U*^11^	*U*^22^	*U*^33^	*U*^12^	*U*^13^	*U*^23^
O1	0.117 (2)	0.1053 (19)	0.0558 (13)	0.0634 (17)	−0.0041 (13)	−0.0040 (12)
O2	0.1006 (18)	0.0891 (16)	0.0580 (13)	0.0258 (14)	0.0307 (12)	0.0011 (11)
O3	0.0851 (17)	0.0709 (15)	0.115 (2)	0.0314 (13)	0.0264 (15)	−0.0126 (14)
O4	0.136 (2)	0.0714 (16)	0.0829 (17)	0.0524 (16)	−0.0107 (16)	0.0009 (13)
N1	0.109 (2)	0.0739 (17)	0.0583 (15)	0.0542 (17)	0.0153 (15)	0.0001 (13)
N2	0.0690 (15)	0.0583 (14)	0.0517 (14)	0.0267 (12)	0.0088 (11)	−0.0026 (11)
N3	0.0658 (15)	0.0538 (14)	0.0513 (14)	0.0095 (12)	0.0085 (12)	−0.0042 (11)
N4	0.0686 (17)	0.0521 (15)	0.083 (2)	0.0200 (13)	−0.0023 (15)	−0.0111 (14)
C1	0.0609 (17)	0.0507 (15)	0.0484 (15)	0.0176 (13)	0.0106 (13)	0.0035 (12)
C2	0.0447 (14)	0.0465 (14)	0.0510 (15)	0.0082 (11)	0.0085 (12)	−0.0016 (11)
C3	0.0482 (15)	0.0464 (14)	0.0459 (15)	0.0056 (12)	0.0063 (11)	−0.0001 (11)
C4	0.0494 (15)	0.0486 (15)	0.0578 (17)	0.0080 (12)	0.0091 (13)	−0.0096 (12)
C5	0.0505 (15)	0.0449 (15)	0.0661 (18)	0.0140 (12)	0.0029 (13)	−0.0031 (13)
C6	0.0649 (18)	0.0569 (17)	0.0542 (17)	0.0183 (14)	0.0027 (14)	0.0034 (13)
C7	0.0661 (18)	0.0586 (17)	0.0473 (15)	0.0211 (14)	0.0073 (13)	−0.0027 (12)

### Geometric parameters (Å, °)

**Table:**

O1—N3	1.214 (3)	C1—H1A	0.9300
O2—N3	1.221 (3)	C2—C7	1.400 (4)
O3—N4	1.229 (3)	C2—C3	1.414 (3)
O4—N4	1.219 (3)	C3—C4	1.382 (3)
N1—N2	1.336 (3)	C4—C5	1.361 (4)
N1—H1B	0.8999	C4—H4A	0.9300
N1—H1C	0.9000	C5—C6	1.393 (4)
N2—C1	1.282 (3)	C6—C7	1.365 (4)
N3—C3	1.464 (3)	C6—H6A	0.9300
N4—C5	1.463 (3)	C7—H7A	0.9300
C1—C2	1.458 (3)		
			
N2—N1—H1B	124.3	C4—C3—C2	122.2 (2)
N2—N1—H1C	115.3	C4—C3—N3	115.3 (2)
H1B—N1—H1C	119.5	C2—C3—N3	122.5 (2)
C1—N2—N1	117.5 (2)	C5—C4—C3	118.9 (2)
O1—N3—O2	121.8 (3)	C5—C4—H4A	120.6
O1—N3—C3	119.9 (2)	C3—C4—H4A	120.6
O2—N3—C3	118.3 (2)	C4—C5—C6	121.7 (3)
O4—N4—O3	123.8 (3)	C4—C5—N4	119.6 (3)
O4—N4—C5	118.6 (3)	C6—C5—N4	118.7 (3)
O3—N4—C5	117.6 (3)	C7—C6—C5	118.4 (3)
N2—C1—C2	118.9 (2)	C7—C6—H6A	120.8
N2—C1—H1A	120.5	C5—C6—H6A	120.8
C2—C1—H1A	120.5	C6—C7—C2	123.2 (3)
C7—C2—C3	115.5 (2)	C6—C7—H7A	118.4
C7—C2—C1	119.4 (2)	C2—C7—H7A	118.4
C3—C2—C1	125.0 (2)		

### Hydrogen-bond geometry (Å, °)

**Table:**

*D*—H···*A*	*D*—H	H···*A*	*D*···*A*	*D*—H···*A*
N1—H1B···O1^i^	0.90	2.52	3.305 (3)	146
N1—H1C···N2^ii^	0.90	2.34	3.123 (4)	146

Symmetry codes: (i) −*x*+2, −*y*, −*z*+1; (ii) −*x*+2, −*y*, −*z*.
